# A Modified Technique for Preventing Lens–Iris Diaphragm Retropulsion Syndrome in Vitrectomized Eyes during Phacoemulsification

**DOI:** 10.3390/jpm13030418

**Published:** 2023-02-26

**Authors:** Zhiyi Wu, Tian He, Zhitao Su, Ye Liu, Jingliang He, Yanan Huo

**Affiliations:** 1 Eye Center, The Second Affiliated Hospital, Zhejiang University School of Medicine, Hangzhou 310010, China; 2 Department of Ophthalmology, The Children’s Hospital of Hangzhou, Hangzhou 310010, China

**Keywords:** modified technique, phacoemulsification, vitrectomized eyes, lens–iris diaphragm retropulsion

## Abstract

Background: Lens–iris diaphragm retropulsion syndrome (LIDRS) is common in vitrectomized or high myopic eyes during phacoemulsification. We evaluated the results of a modified technique for cataract treatment using phacoemulsification in vitrectomized eyes. Methods: In this retrospective study, we enrolled thirty-four vitrectomized eyes treated with modified phacoemulsification (Modified Group) and nineteen vitrectomized eyes treated with routine phacoemulsification (Control Group). The modified technique comprised irrigation with a balanced salt solution underneath the pupil before phacoemulsification instrument entry, lens implantation and stromal hydration to stabilize the anterior chamber and equilibrate the pressure between the anterior chamber and posterior cavity. Results: We compared the incidences of intra and postoperative complications and visual outcomes between modified and routine phacoemulsification. Pain, LIDRS and difficulty in stromal hydration were significantly more common in the Control Group than in the Modified Group (*p* < 0.05). There were no significant differences in the rates of posterior capsular rupture, iris trauma, lens dislocation, or posterior capsular opacification between the Modified and Control Groups (*p* > 0.05). However, there was no significant difference in visual acuity between the groups (*p* > 0.05). Complications such as loss of nuclear fragments into the vitreous cavity, cystoid macular edema, retina redetachment, suprachoroidal hemorrhage and vitreous hemorrhage did not occur either intra or postoperatively in any of our patients. Conclusions: Our modified technique prevents LIDRS and complications arising during cataract surgery in vitrectomized eyes. Aside from this, the results of modified and routine phacoemulsification are similar in vitrectomized eyes.

## 1. Introduction

A cataract is an eye disease that involves the opacification of the crystalline lens of the eye or its envelope. There are many known causes of cataracts, including the natural aging process, nutritional disorders, metabolic abnormalities such as diabetes, chronic ocular inflammation and certain injuries. Intraocular surgery is the gold standard for cataract surgery. But it can format or accelerate cataracts, especially pars plana vitrectomy (PPV), which is a microsurgical technique to treat certain disorders affecting the posterior segment of the eyes. During vitrectomy surgery, three small incisions are made in the eye in order to place the following instruments: a fiberoptic light source to illuminate the inside of the eye, a vitreous cutter, and an infusion cannula to maintain proper intraocular pressure during the surgery. Advances in PPV surgical techniques and instrumentation have revolutionized the treatment of posterior segment disorders. However, there remain some surgical risks of significant vision loss, including retinal detachment, corneal endothelial decompensation and cataract formation or progression in phakic eyes. Nuclear sclerotic cataract development is the most frequent complication after pars plana vitrectomy (PPV) in the phakic eye [1,2]. However, phacoemulsification is a challenge in the vitrectomized eye because of the lack of vitreous support, the unstable anterior chamber depth and the density of the nuclear cataract. In addition, intraoperative complications such as intraoperative ocular pain and lens dislocation can also increase the difficulty of surgical procedures. Additionally, the risk and incidence of complications are higher in cataract surgery after a previous PPV than in non-PPV eyes. Hence, phacoemulsification in the vitrectomized eye is associated with higher rates of intra and postoperative complications [3,4,5,6,7,8].

Lens–iris diaphragm retropulsion syndrome (LIDRS) was first described in 1992 by Zauberman and further named by Wilbrandt and Wilbrandt in 1994 [9,10,11,12,13,14,15,16,17,18,19]. The incidence rate of LIDRS in vitrectomized eyes during phacoemulsification widely varies, ranging from 4.5% to 100% [11,12]. The syndrome is characterized by the anterior chamber (AC) deepening, followed by pupil dilation and a typical concave iris configuration. During cataract surgery, as the initial corneal incision is made, fluid is lost from both the AC and the vitreous cavity, resulting in the loss of AC and vitreous body volumes. When phacoemulsification or irrigation/aspiration (I/A) probes are inserted, the irrigation causes significant differences in the pressure between the anterior and posterior compartments. The AC deepens, and the iris bows posterior to the lens, blocking fluid passage from the AC. The surgeon must adjust the operative plane by positioning the instruments deeper, which may deform the incision and compromise performance. During phacoemulsification or cortical aspiration, the probe is positioned posterior to the iris plane, resulting in changes in fluid dynamics. Fluid may enter the vitreous cavity through zonular defects, increasing posterior cavity pressure and causing shallowing of the AC and miosis. IDS can lead to complications, such as posterior capsular rupture (PCR), iris trauma, expulsive choroidal hemorrhage and choroidal detachment.

The literature on LIDRS prevention during cataract surgery in vitrectomized eyes is scarce [6]. To minimize LIDRS and LIDRS-related complications, we modified the technique by irrigating a balanced salt solution (BSS) into the vitreous cavity through a syringe with a bent, blunt-tipped needle. This technique stabilized the AC intraoperatively, preventing abrupt excursions of the iris–lens diaphragm and making emulsification, cortical aspiration, intraocular lens (IOL) implantation and stromal hydration safer.

In this retrospective study, we compared the intra and postoperative complications of the modified (Modified Group) and routine (Control Group) phacoemulsification surgeries in eyes after 23-gauge PPV.

## 2. Materials and Methods

### 2.1. Participants

We retrospectively reviewed the medical records of 59 patients (62 eyes) who underwent consecutive phacoemulsification and IOL implantation after a previous 23-gauge three-port PPV surgery between 12 January 2015 and 31 December 2016 in the Eye Center of the Second Affiliated Hospital of Zhejiang University School of Medicine. Patients with in situ silicone oil were excluded from the study. This study followed the tenets of the *Declaration of Helsinki*. The Ethics Committee of the Second Affiliated Hospital of Zhejiang University School of Medicine approved this study (No. 2015-003).

Data were gathered on age, sex, indications for PPV, indications of PPV, preoperative evaluations, intraoperative observations and complications. The patients were followed-up postoperatively on day 1, as well as 1, 2, 4, 8, 12 and 24 weeks after their cataract surgery. Visual acuity and intraocular pressure assessments and slit-lamp examinations were performed, and any postoperative complications were noted. Neodymium-yttrium-aluminum-garnet (Nd: YAG) laser posterior capsulotomy was performed for residual posterior capsular opacification.

### 2.2. Surgical Techniques

All vitrectomy and cataract surgeries were conducted by a single experienced surgeon using the associate vitrectomy and phacoemulsification machine (Stellaris PC; Bausch + Lomb: Rochester, NY, USA). All the patients underwent cataract surgery under topical anesthesia (proparacaine hydrochloride 0.5%).

In the Control Group, a side port corneal incision was created with a blade initially. A small amount of viscoelastic was then injected into the anterior chamber before the 2 mm superior corneal incision was made. A continuous curvilinear capsulorrhexis was made under viscoelastic in all eyes. Hydrodissection was performed using a BSS supplemented with adrenaline. The nucleus was chopped using the “phaco-chop” or “stop-and-chop” methods. A 30° phacoemulsification probe was used for all the patients. Cortical clean-up was performed using an I/A probe. A foldable IOL (CT ASPHINA 509M; Carl Zeiss Meditec: Jena, Germany/iSert^®^ 250; Hoya Surgical Optics Inc.: Chino Hills, CA, USA) was implanted into the capsular bag. Any residual viscoelastic material was completely removed from the AC and behind the IOL with an I/A probe. The stromal hydration of the side port and main incision was performed using the BSS. At the end of the procedure, a 0.1% TobraDex^®^ ointment (Alcon Laboratories, Inc.: Fort Worth, TX, USA) was administered.

**Figure 1:**
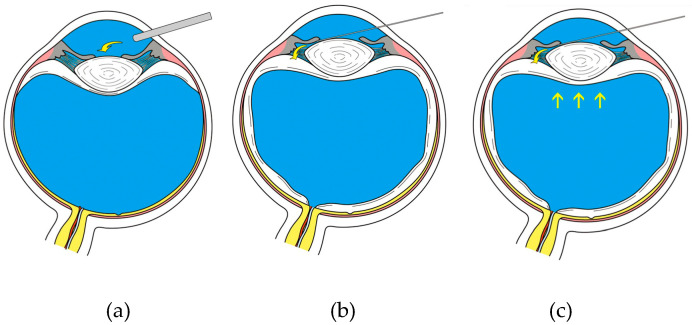
Images demonstrating intraocular fluidics in vitrectomized eyes before and after the modified technique. (**a**) As the irrigating phacoemulsification probe enters the anterior chamber, the iris-lens diaphragm is displaced posteriorly, creating a relative pupillary block. (**b**) Lens–iris diaphragm retropulsion syndrome controlled by lifting the iris with the needle and irrigating a balanced salt solution underneath the iris, allowing flow of fluid into the vitreous cavity. (**c**) This technique stabilizes the iris-lens diaphragm as the pressure equilibrates.

**Figure 2:**
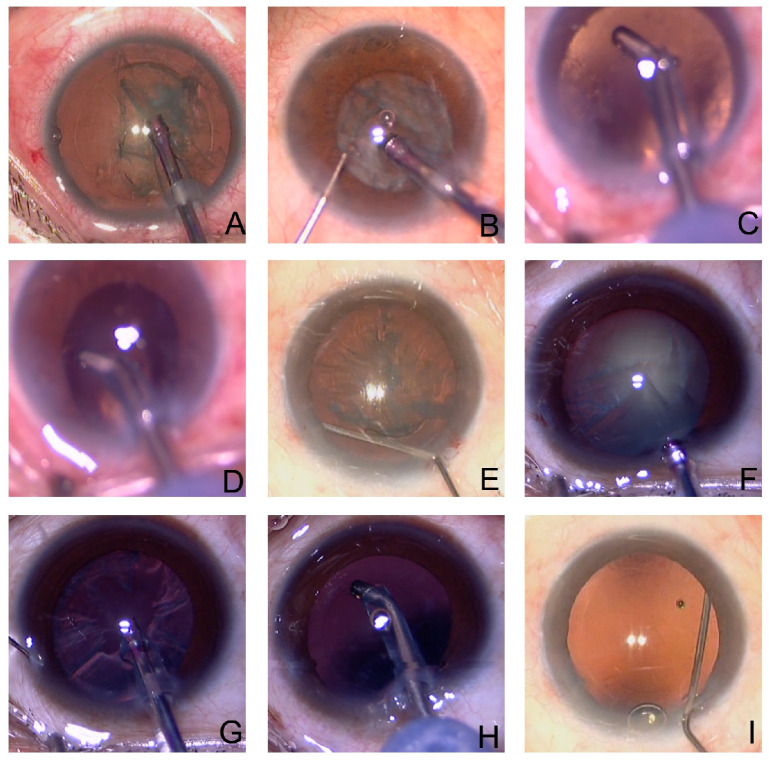
Photographs demonstrating our surgical technique to manage infusion deviation syndrome. (**A**) When the phacoemulsification probe is first inserted, the anterior chamber (AC) deepens, and pupil size increases in Control Group. (**B**) The changes in fluid dynamics cause shallowing of the AC, with miosis following immediately during emulsification in the Control Group. (**C**,**D**) The same phenomena (infusion deviation syndrome) occur during cortical aspiration. (**E**) Before emulsification, a BSS is irrigated underneath the pupil to stabilize the AC in the Modified Group. (**F**,**G**) With the modified irrigation technique, the AC depth remains stable during emulsification procedures in the Modified Group. (**H**) The AC depth remains stable during cortical aspiration in the Modified Group. (**I**) The modified technique performed before IOL insertion.

The main steps of the modified technique, including phacoemulsification, I/A, IOL implantation and stromal hydration, were the same as those of routine cataract surgery. However, to stabilize the AC, we irrigated the BSS underneath the pupil using a syringe with a bent, blunt-tipped needle for a few seconds, which allowed the fluid to enter the vitreous cavity (Figure 1). This irrigation was performed before phacoemulsification, I/A probe entry, IOL insertion and stromal hydration to enable pressure equilibration between the anterior and posterior cavities, to prevent abrupt excursions of the iris–lens diaphragm and to facilitate sculpting and nuclear fragmentation (Figure 2).

The surgeries were recorded using a video system and analyzed for both groups. The settings for phacoemulsification (Stellaris PC) were as follows: phacoemulsification power, 0–50% (depending on the grade of the nucleus), and vacuum limit, 350 mmHg. The bottle height was 90 cm above the patient’s head.

### 2.3. Statistical Analysis

SPSS 23.0 software (IBM: Armonk, New York, NY, USA) was used for all statistical analyses. Data were presented as the mean ± standard deviation or as *n* (%) for categorical variables. We used Student’s *t*-test for normally distributed variables, the Kruskal–Wallis test for non-parametric variables, and the chi-squared or Fisher’s exact tests, as indicated for the analyses of categorical variables. Snellen’s best-corrected visual acuity measurements were converted to the logarithm of the minimum angle of resolution (logMAR) equivalents for the purpose of data analysis. A *p*-value of <0.05 was considered statistically significant.

## 3. Results

This retrospective study enrolled 59 patients (62 eyes). Seven patients (nine eyes) with in situ silicone oil were excluded from this study. The mean age of the patients in the Modified Group was 64.03 ± 13.12 years, whereas that of patients in the Control Group was 57 ± 12.31 years (*p* > 0.05). There were twenty-one male and thirteen female patients in the Modified Group and thirteen male and six female patients (19 eyes) in the Control Group (*p* > 0.05). The interval between PPV and phacoemulsification was not significantly different between the Modified (8.39 ± 4.7 months) and Control (9.9 ± 5.22 months) Groups.

Table 1 summarizes the indication for PPV. The indications for PPV in the Modified Group were retinal detachment in 18/34 eyes (52.9%), macular hole in 5/34 eyes (14.7%), proliferative diabetic retinopathy in 5/34 eyes (14.7%), epiretinal membrane in 4/34 eyes (11.8%) and retinal vein occlusion in 2/34 eyes (5.9%). In contrast, in the Control Group, the indications for PPV were retinal detachment in 8/19 eyes (42.1%), a macular hole in 5/19 eyes (26.3%), proliferative diabetic retinopathy in 3/19 eyes (15.8%), retinal vein occlusion in 2/19 eyes (10.5%) and epiretinal membrane in 1/19 eyes (5.3%; Table 1). The indications for PPV were not significantly different between the Modified Group and Control Group (all *p* > 0.05).

Table 2 summarizes the intraoperative observations and complications encountered. Not all vitrectomized eyes developed LIDRS in our study. Characteristically, the anterior chamber depth appeared abnormal as soon as irrigation commenced. The iris-lens diaphragm bowed posteriorly, causing the anterior chamber to deepen excessively and the pupil to dilate widely. During nuclear sculpting, the deepening of the abnormal anterior chamber necessitated steeper angulation of the phacoemulsification probe, and the nucleus was also noted to be more mobile than usual. Intraoperatively, LIDRS was noted in 2/34 eyes (5.9%) in the Modified Group and in 8/19 eyes (42.1%) in the Control Group (*p* < 0.05; Table 2). Patients with 11/19 eyes (57.9%) in the Control Group complained of sudden pain (in the range of 2–3 out of 10 on the numerical rating scale) when instruments entered the AC, whereas none of the 34 patients in the Modified Group felt pain (*p* < 0.05). In the Control Group, 1/19 eyes (5.3%) had PCR, and the IOL was placed in the ciliary sulcus. Additionally, in the Control Group, 2/19 eyes (10.5%) developed iris trauma during aspiration of the cortex and viscoelastic material. However, IOL dislocation occurred in only 1/34 eyes (2.9%) in the Modified Groupand was replaced in the capsular bag immediately. Difficulty in stromal hydration was observed in 4/34 eyes (11.8%) in the Modified Group and 7/19 eyes (36.8%) in the Control Group (*p* < 0.05). Cataract surgery was completed in all cases, in both the Modified Group and the Control Group.

No complications, such as loss of nuclear fragments into the vitreous cavity, cystoid macular edema, retinal redetachment, suprachoroidal hemorrhage, or vitreous hemorrhage, occurred either intra or postoperatively in any of the patients. Posterior capsular opacification was evident in 9/34 eyes (26.5%) in the Modified Group and in 5/19 eyes (26.3%) in the Control Group (*p* = 0.99); this was successfully removed using the Nd: YAG laser. No patients in either group required any surgical intervention in the 24-week follow-up period (Table 2).

The final visual outcomes were dictated by the nature of the retinal pathology present at the time of the initial vitrectomy procedure. The mean preoperative best-corrected visual acuity of the Modified Group was 1.11 ± 0.46 logMAR units, and that of the Control Group was 1.13 ± 0.52 logMAR units. Both improved significantly at 24 weeks after surgery, to 0.58± 0.35 logMAR units in the Modified Group and 0.59 ± 0.43 logMAR units in the Control Group (both *p* < 0.005). The final visual acuity was similar between the groups (*p* > 0.05). External segment, ocular motility, pupillary function and intraocular pressure were within normal limits in both groups.

## 4. Discussion

PPV was first developed by Machemer in 1971. It is an effective, small-gauge, safe surgery that is essential in the treatment of a variety of posterior segment pathologies, including retinal detachment, macular hole, proliferative diabetic retinopathy, vitreous hemorrhage due to diabetic retinopathy or vein occlusion, preretinal membrane and endophthalmitis. However, performing a vitrectomy can induce cataracts, particularly with the use of intraocular gas, even in young patients. Cataracts eventually occur in almost all eyes after PPV [13]. The causative factors of cataract formation or acceleration after PPV have been linked with the use of intraocular gas, oxidation of lens protein, light toxicity, length of operative time and oxygen tension within the eye. In non-vitrectomized eyes, the vitreous body (especially the vitreous base) limits the flow of fluid from the posterior chamber into the vitreous cavity, preventing changes in volume and pressure in both the AC and posterior segment. However, phacoemulsification in vitrectomized eyes is more technically challenging than that in nonvitrectomized eyes [14]. The primary reason for this is the loss of vitreous counterpressure in vitrectomized eyes. Some vitrectomized eyes also have localized zonular weaknesses caused by loss of the vitreous scaffold, stretching of the zonules by expansile gas or oil, or damage to the zonular apparatus during vitrectomy [15]. This may lead to faster and easier fluid exchange between the AC and posterior cavity. Extremely stretched zonules can be easy to break, causing lens dislocation. Additionally, strong fluctuations in the AC or the iris–capsular bag diaphragm may cause patients pain and trigger unexpected abrupt agitation, increasing the difficulty of the surgery [16]. The nucleus tends to be harder than in age-related nuclear sclerosis, requiring longer phacoemulsification time during the procedure. Together, the unstable posterior capsule and zonules, extended operative time and increased pain experienced by the patients increase the likelihood of complications such as expulsive choroidal hemorrhage or choroidal detachment. With the increasing use of vitrectomy in the treatment of various posterior segment disorders, we expect to see an increase in the number of such cataracts being referred to general ophthalmologists and anterior segment surgeons. Unfortunately, most complications are unpredictable. Specific surgical experience and skills related to the management of complications during cataract surgery in vitrectomized eyes are required. 

Many studies have reported that phacoemulsification is surgically more challenging in vitrectomized eyes than in nonvitrectomized eyes because various anatomic changes within the eye confer a higher risk of complications. In 1992, Zauberman [8] first described the phenomenon of AC deepening, excessive pupil dilation and a concave shape of the iris during phacoemulsification. Wilbrandt and Wilbrant further studied this syndrome and named it LIDRS in 1994 [7]. LIDRS are more likely to happen in eyes that have had multiple or extensive PPV for diabetic proliferative retinopathy and retinal detachment. However, less LIDRS or abnormally deep AC was evident in eyes that had undergone a limited “core vitrectomy”, such as for a macular hole or epiretinal membrane. In our study, of the ten patients noted to develop LIDRS, eight had undergone thorough PPV for retinal detachment or diabetic proliferative retinopathy. These eight patients previously had thorough vitreous removal through peripheral indentation and trimming of the vitreous base in order to relieve anterior vitreous traction. These procedures may have caused structural damage in the vitreous base region, resulting in abnormal laxity of the zonules. Szijarto et al. [17] observed a deep or fluctuating AC in 93% of vitrectomized eyes, implying that the occurrence of LIDRS is related to the loss of the vitreous body, especially the vitreous base. Other studies have reported that the rate of LIDRS-related complications, such as PCR during cataract surgery in vitrectomized eyes, ranges from 0 to 11.4% [6,7,17,18,19,20,21,22,23]. The rate of dropped nuclei ranges from 0 to 4.5% [6,7,17,18,19,20,21,22,23,24], while the rate of zonular dialyses ranges from 0 to 5% [6,7,17,18,19,24], and the rate of iris trauma ranges from 0 to 0.2% [20,21,24]. The rate of posterior capsular opacification requiring Nd: YAG laser capsulotomy after cataract surgery reportedly ranges from 2.2 to 44% in vitrectomized eyes, depending on the follow-up period [6,17,18,19,24]. The rate of retinal detachment in the early postoperative period after cataract surgery in vitrectomized eyes ranges from 1.2 to 6% [17,18,19,24]. The rate of decentration and dislocation of the IOL is around 2 to 2.9% [18,22], whilst the rate of hypotony with choroidal effusion ranges from 0 to 0.6% [18,20,21], and the rate of vitreous hemorrhage ranges from 0.6 to 6% [18,19]. Valesová L et al. reported in 2004 that the incidence of intraoperative complications in the posterior perfusion cataract surgery was slightly higher than in the standard cataract surgery group; there were no special complications in the standard cataract surgery group. Furthermore, the safety of the two surgical methods was consistent [25]. In this study, our findings were significantly different from the study by Valesová L et al. in 2004, as the method in our study differs from theirs. Our method is simple, fast, safe, non-invasive and does not require additional equipment. However, their study used an invasive method to create a new perfusion hole in the eye, which would increase the eyeball damage, the length of the operation and the back pressure, resulting in other complications. In addition, their study showed that their invasive approach does not reduce the risk of surgery. 

In contrast, we experienced significantly fewer complications during cataract surgery using the modified technique. Pain upon the entry of irrigation into the AC did not occur in the Modified Group; however it was reported by 57.9% of the Control Group (*p* < 0.05). The rates of LIDRS and iris trauma were also significantly lower in the Modified Group than in the Control Group (both *p* < 0.05). 

Immediately closing corneal incisions after surgery is important for preventing postoperative complications such as hypotony, choroidal effusion and endophthalmitis. However, stromal hydration can be hindered by ocular hypotony during surgery in vitrectomized eyes. Sachdev et al. [20] reported a rate of early postoperative hypotony and serous choroidal detachment of 1.3% after cataract surgery in vitrectomized eyes. Other authors have reported the use of 10/0 sutures to seal the corneal incision [18]. However, in our study, irrigation with a BSS before sealing the incisions seemed sufficient for solving this problem and preventing related complications. The rate of difficulty in sealing the corneal incision was significantly lower in the Modified Group than in the Control Group (*p* < 0.05). 

One patient (2.9%) in the Modified Group experienced IOL dislocation caused by excessive irrigation during stromal hydration. The IOL was repositioned, and relatively good visual performance was attained without further treatment. 

Patients who develop cataracts after PPV surgery may undergo a phacoemulsification cataract surgery. Although visual acuity in a normal eye typically improves after cataract surgery, the visual prognosis after surgery for post vitrectomy cataract may be uncertain. Visual acuity and other outcomes after phacoemulsification cataract surgery in eyes undergoing vitrectomy are dependent on multiple factors, although primarily on the retinal condition and the avoidance of complications during cataract surgery. In our experience, the patients who had undergone PPV for macula-on retinal detachment or a macular hole experienced a better improvement in visual acuity after cataract surgery. The patients who had undergone PPV for macula-off retinal detachment or proliferative diabetic retinopathy experienced less visual improvement. There were no severe complications, such as dropped nuclei, expulsive choroidal hemorrhage or choroidal detachment, in the present study. The number of eyes that received Nd: YAG laser treatment after cataract surgery did not differ between the two groups in this study; this confirms the safety of the modified procedure.

The timing of irrigation in our modified technique is important. As both the AC and vitreous body fluid are continually lost from the corneal incision, we balanced the pressure between the AC and posterior segment by irrigation of a BSS underneath the iris at four time points:

(1) Before the first entry of the phacoemulsification probe;
(2) Before the first entry of the I/A probe;
(3) Before the insertion of the IOL; and
(4) Before the hydration of the stromal incision.

This modified irrigation regimen allows fluid to enter the posterior segment, resulting in pressure equilibration. Using the above steps not only made operating on these eyes safer, since the phaco handpiece could be held in a normal position, but it also greatly enhanced the patient’s comfort. As shown in Table 2, this irrigation significantly decreased the incidences of pain, LIDRS, PCR and difficulty in stromal hydration (all *p* < 0.05). 

In our study, all of the patients had phacoemulsification cataract surgery through a superior corneal incision instead of a scleral tunnel. In our opinion, it is preferable to have a steeper angulation of the phacoemulsification probe when operating on vitrectomized eyes. Using a corneal incision also avoids conjunctival scarring or postoperative infection resulting from occult filtration.

In our experience, LIDRS does not only occur in phakic eyes after PPV; it also occurs in eyes with high myopia as a result of synchysis, greater AC and axial lengths, a floppy posterior capsule and zonular laxity. High myopia eyes with elongated stretched zonular fibers are more prone to develop LIDRS during phacoemulsification cataract surgery. Additional techniques can be used in vitrectomized and high myopic eyes to minimize IDS, such as reducing the height of the liquid bottle to about 90 cm, using a low-flow rate and vacuum limit, and using a finger to press the corneal incision. A longer, better self-sealing corneal incision and a shorter interval between each step may decrease fluid loss and intraocular pressure fluctuation, meaning that the AC may remain watertight.

Other authors have advocated different solutions for stabilizing the AC, such as forming the AC with viscoelastic material during the routine phacoemulsification procedure [2]. An AC maintainer and an irrigating chopper have also been reported to prevent AC fluctuation [26,27]; however, an AC maintainer requires an additional corneal incision. The use of an irrigating chopper in microincisional cataract surgery in tandem with cold phacoemulsification technology has been reported; however, the issue of wound burns persists because of the “naked” phacoemulsification needle [3,6,28]. Joshi [29] modified the sleeve of a phacoemulsification probe to increase fluid flow and deepen the capsular bag, with the aim of decreasing the fluctuation in the AC. However, both prospective and retrospective comparative studies on the prevention of LIDRS in vitrectomized eyes after cataract surgery are lacking [7]. Our study had certain limitations. There are significant astigmatic changes during the early postoperative period and posterior capsular transparency changes during the late postoperative period. Continued follow-ups of the patients are necessary to monitor the long-term refractive stability and visual acuity of these procedures.

## 5. Conclusions

In summary, our time-saving technique has demonstrated good surgical results in this retrospective study. This simple technique increases fluid flow into the vitreous cavity, resulting in pressure equilibration and a reduced risk of complications without using additional instruments during routine cataract surgery. The rate of LIDRS and related complications were relatively low compared with the Control Groupand the findings of previous studies. Therefore, we recommend considering this approach in patients with a history of PPV.

## Figures and Tables

**Table 1:** Indications for pars plana vitrectomy (PPV) in the Modified and Control Groups.

Indication for PPV	Total Number of Eyes (*n* = 53)		
	Modified Group (*n* = 34)	Control Group (*n* = 19)	*p* Value
Retinal detachment	18 (52.9%)	8 (42.1%)	0.45
Macular hole	5 (14.7%)	5 (26.3%)	0.3
Proliferative diabetic retinophathy	5 (14.7%)	3 (15.8%)	0.922
itreous hemorhage	4 (11.8%)	2 (10.5%)	0.89
piretinal membrane	2 (5.9%)	1 (5.3%)	0.93

Table 1 was analyzed using chi-square tests, with continuity correction and Fisher’s Exact Test.

**Table 2:** Intraoperative and postoperative observations and complications encountered.

Complication	Total Number of Eyes (*n* = 53)		
	Modified Group (*n* = 34)	Control Group (*n* = 19)	*p* Value
Intraoperative			
LIDRS	2 (5.9%)	8 (42.1%)	0.001 *
Pain	0 (0%)	11 (57.9%)	<0.001 *
Posterior capsular rupture	0 (0%)	1 (5.3%)	0.177
Iris trauma	0 (0%)	2 (10.5%)	0.054
Dislocation of IOL	1 (2.9%)	0 (0%)	0.45
Difficulty in stromal hydration	4 (11.8%)	7 (36.8%)	0.03 *
Postoperative			
PCO requiring Nd: YAG laser capsulotomy	9 (26.5%)	5 (26.3%)	0.99

Table 2 were analyzed by chi-square tests, with continuity correction and Fisher’s Exact Test. * *p* < 0.05, statistically significant.

## Data Availability

Not applicable.
